# Tumor Necrosis Factor-Alpha (TNF-α) Levels in Women With Polycystic Ovary Syndrome (PCOS): A Systematic Review and Meta-Analysis of Observational Studies

**DOI:** 10.7759/cureus.99677

**Published:** 2025-12-19

**Authors:** Bhanu Verma, Pranav Verma, Sreelakshmi S Nair, Sophia John Bosco, Sasikala Kathiresan, Abhishek Hanumanpratap Singh Kshatri, Animesh Kumar Tiwari, Amrutha R Kenche, Parag Bashichandra, Sangeeth Kumar Indu Kumar, Delna NS, Akshay V P, Shubhrit Shrivastava

**Affiliations:** 1 Pharmacy, University School of Pharmaceutical Sciences, Rayat Bahra University, Kharar, IND; 2 Pharmacy, Chitkara College of Pharmacy, Chitkara University, Rajpura, IND; 3 Genetics, Sri Ramaswamy Memorial (SRM) Institute of Science and Technology, Chennai, IND; 4 Biochemistry, Faculty of Medicine, Sri Lalithambigai Medical College and Hospital, Dr. M.G.R. Educational and Research Institute, Chennai, IND; 5 Obstetrics and Gynecology, All India Institute of Medical Sciences, Madurai, IND; 6 Emergency Medicine, Apollo Hospitals, Visakhapatnam, IND; 7 Forensic Science, Guru Ghasidas Vishwavidyalaya, Bilaspur, IND; 8 Biosciences and Bioengineering, D. Y. Patil International University (DYPIU), Pune, IND; 9 Bioscience and Biomedical Engineering, Indian Institute of Technology, Bhilai, IND; 10 Biochemistry, Srinivas University, Mangaluru, IND; 11 Allied Health Sciences, Al-Azhar Medical College and Super Speciality Hospital, Thodupuzha, IND; 12 Biotechnology, Mansarovar Global University, Bhopal, IND; 13 Molecular Biology, BioDesk India Labs, Bhopal, IND; 14 Biochemistry, BioDesk India Labs, Bhopal, IND

**Keywords:** meta-analysis, pcos, proinflammatory cytokine, systematic review, tumor necrosis factor-alpha

## Abstract

Tumor necrosis factor-alpha (TNF-α) is a proinflammatory cytokine. Evidence relating circulating TNF-α concentrations to polycystic ovary syndrome (PCOS) is heterogeneous and has not been synthesized comprehensively for clinical interpretation. We aim to systematically review and meta-analyze observational studies comparing circulating TNF-α levels between women with PCOS and healthy controls, and to examine sources of between-study heterogeneity and the robustness of the findings.

We conducted a systematic search of electronic databases following Preferred Reporting Items for Systematic Reviews and Meta-Analyses 2020 guidance. Eligible studies reported serum TNF-α concentrations in reproductive-age women with PCOS and matched controls. Two reviewers independently screened records, extracted data, and assessed risk of bias. Where appropriate, we pooled continuous outcomes using random-effects meta-analysis (standardized mean difference (SMD)) with restricted maximum likelihood τ² estimation and Hartung-Knapp adjustment. Prespecified subgroup and meta-regression analyses explored effects of assay type, body mass index (BMI) matching, diagnostic criteria (Rotterdam vs. other), and geographic region. Small-study effects were assessed using contour-enhanced funnel plots, Egger’s test, and selection-model sensitivity analyses.

Overall, circulating TNF-α was modestly higher in women with PCOS than in controls (SMD = 0.48; 95% CI = 0.17-0.79; p = 0.0026). Between-study heterogeneity was substantial (I² = 89.5%, τ² = 0.2772). Funnel-plot inspection and Egger’s test showed no clear small-study bias (t = -0.96; p = 0.36); trim-and-fill imputed four studies and produced a larger adjusted SMD (0.80; 95% CI = 0.44-1.16), but heterogeneity remained high. Key subgroup findings include the following: 1) obese cohorts (k = 11) showed a significant but small effect (SMD = 0.39; 95% CI = 0.09-0.69), whereas evidence in lean cohorts (k = 2) was inconclusive (SMD = 0.97; 95% CI = -0.23 to 2.17); 2) by assay, ELISA studies (k = 10) were significant (SMD = 0.50), and chemiluminescence studies (k = 2) were consistent (SMD = 0.42, I² = 0%); and 3) country of origin materially moderated effects (Q = 53.17, p < 0.0001), with larger effects in studies from India and China and null findings in some Iranian studies. Leave-one-out sensitivity analyses produced pooled SMDs of 0.39-0.55. Meta-regression for mean BMI, age, and sample size did not explain heterogeneity.

In conclusion, circulating TNF-α concentrations were higher in women with PCOS than in healthy controls; this finding is biologically plausible and consistent across studies. These findings suggest TNF-α may serve as an inflammatory biomarker reflecting metabolic dysregulation in PCOS.

## Introduction and background

Polycystic ovary syndrome (PCOS) is a complex and heterogeneous endocrine disorder affecting approximately 5%-15% of women of reproductive age [1]. It presents with a spectrum of metabolic, reproductive, and psychological features, including hyperandrogenism, ovulatory disruption, polycystic ovarian morphology, insulin resistance, obesity, and elevated cardiovascular risk [2,3].

Chronic low-grade inflammation has become recognized as a central contributor to PCOS-related metabolic and reproductive dysfunction. In PCOS patients, studies consistently show elevated systemic inflammatory markers, which are believed to exacerbate insulin resistance, dyslipidemia, endothelial impairment, and cardiovascular complications. In the ovarian milieu, inflammation can disrupt follicular growth, steroid synthesis, and oocyte quality, further aggravating infertility and menstrual irregularities [4,5].

Among proinflammatory mediators, tumor necrosis factor-alpha (TNF-α) is especially important due to its dual role in metabolic and reproductive pathways. TNF-α plays an important mechanistic role in several pathways implicated in PCOS. In metabolic tissues, TNF-α interferes with insulin receptor signaling by promoting serine phosphorylation of insulin receptor substrate-1, a change that reduces downstream insulin action and contributes to insulin resistance, a core metabolic feature of PCOS. In adipose tissue, TNF-α is produced by hypertrophic adipocytes and resident macrophages, amplifying the chronic low-grade inflammatory state observed in many women with PCOS. Within the ovary, TNF-α modulates steroidogenesis by altering theca-cell androgen production and disrupting follicular maturation. Together, these effects provide a biologically plausible basis for examining circulating TNF-α levels as a marker of inflammation and metabolic dysfunction in PCOS [3-5].

A prior meta-analysis by Gao et al. concluded that circulating TNF-α levels are significantly higher in women with PCOS compared with healthy controls, but methodological heterogeneity limited interpretability [5].

Discrepancies across studies may stem from differences in diagnostic criteria (Rotterdam vs. AES), adiposity confounding (especially obesity), assay variation, and ethnic/regional inflammatory phenotypes. Obesity, per se, induces TNF-α secretion, complicating attribution of elevated inflammation specifically to PCOS.

While several reviews have surveyed inflammation in PCOS broadly, few have undertaken quantitative meta-analysis focusing on TNF-α with robust exploration, and despite consistent elevation of inflammatory markers, TNF-α’s clinical interpretability as a biomarker for PCOS severity or phenotype differentiation remains uncertain [2-6]. To address these gaps, the present systematic review and meta-analysis synthesizes studies between 2014 and 2025 comparing circulating TNF-α levels in PCOS vs. control subjects. By clarifying the consistency and magnitude of association, this review intends to inform understanding of TNF-α’s role in PCOS pathophysiology and direct future biomarker and therapeutic strategies. We hypothesized that circulating TNF-α is elevated in PCOS and that body mass index (BMI), assay variability, and regional differences contribute to heterogeneity.

Materials and methods

This systematic review was conducted following the Preferred Reporting Items for Systematic Reviews and Meta-Analyses (PRISMA) 2020 guidelines. The protocol was prospectively registered in the International Prospective Register of Systematic Reviews (CRD420251044690), and the full protocol and associated data related to the review are available in Open Science Framework [7].

Inclusion and Exclusion Criteria

Studies were selected according to the Population, Exposure, Comparator, Outcome, and Study-design framework: 1) Population (P): Reproductive-age women diagnosed with PCOS using standardized diagnostic criteria such as Rotterdam 2003, NIH 1990, or Androgen Excess Society (AES) 2006; 2) Exposure/Index (I/E): Presence of PCOS (case group); 3) Comparator (C): Healthy women without PCOS (control group). Preference was given to studies with age- and BMI-matched controls; studies without explicit matching were eligible if BMI was reported and/or if adjusted analyses were presented; and 4) Outcome (O): Circulating TNF-α concentrations measured in serum or plasma. Eligible reports provided extractable quantitative data (means ± standard deviations (SDs)) or reported medians/interquartile ranges (IQRs) with sufficient information to estimate means and SDs.

We limited inclusion to studies published since January 2014. A comprehensive prior meta-analysis by Gao et al. [5] synthesized all available studies on TNF-α in PCOS up to July 2016, incorporating 29 primary investigations published between 1999 and 2015. Because this earlier work had already consolidated the entire pre-2014 literature, we aimed to produce an updated evidence synthesis from the most recent decade. Restricting the search to post-2014 studies prevents redundancy with the earlier meta-analysis and ensures that the current review functions as a methodological and chronological extension rather than a duplication of prior work.

Study design: Observational studies (cross-sectional, case-control, cohort baseline comparisons) from hospital-based or community-based settings and peer-reviewed original articles in English published between January 1, 2014, and August 2025 that compared circulating TNF-α levels in women with PCOS and non-PCOS controls and provided extractable quantitative data were included.

Exclusion criteria: Reviews, editorials, conference abstracts, case reports, animal or in vitro studies, studies without a control group, non-peer-reviewed reports, studies not in English, and studies lacking extractable TNF-α data after attempted contact with authors or after planned conversions were excluded.

Literature Search Strategy

A comprehensive literature search was conducted in scholarly databases, including PubMed/MEDLINE, Web of Science, Scopus, and the Cochrane Library. Additional searches were performed in Google Scholar to capture gray literature and cross-references from bibliographies of included studies. Final database search conducted on August 20, 2025. The search strategy combined Medical Subject Headings (MeSH) and free-text terms using Boolean operators. Examples of key terms included “Polycystic Ovary Syndrome”, “PCOS”, “Tumor Necrosis Factor-alpha”, “TNF-α”, and “Inflammation”. Search results were managed using the reference management software, and duplicate records were removed.

Query Strings

("Polycystic Ovary Syndrome"[Mesh] OR "PCOS") AND ("Tumor Necrosis Factor-alpha"[Mesh] OR "TNF-alpha" OR "TNF-α") AND (levels OR concentration) AND (observational OR "cross-sectional" OR "case-control" OR cohort) "Polycystic Ovary Syndrome" OR "PCOS" "Tumor Necrosis Factor-alpha" OR "TNF-alpha" OR "TNF-α" levels concentration observational "cross-sectional" "case-control" cohort.

Study Selection

**Figure 1:**
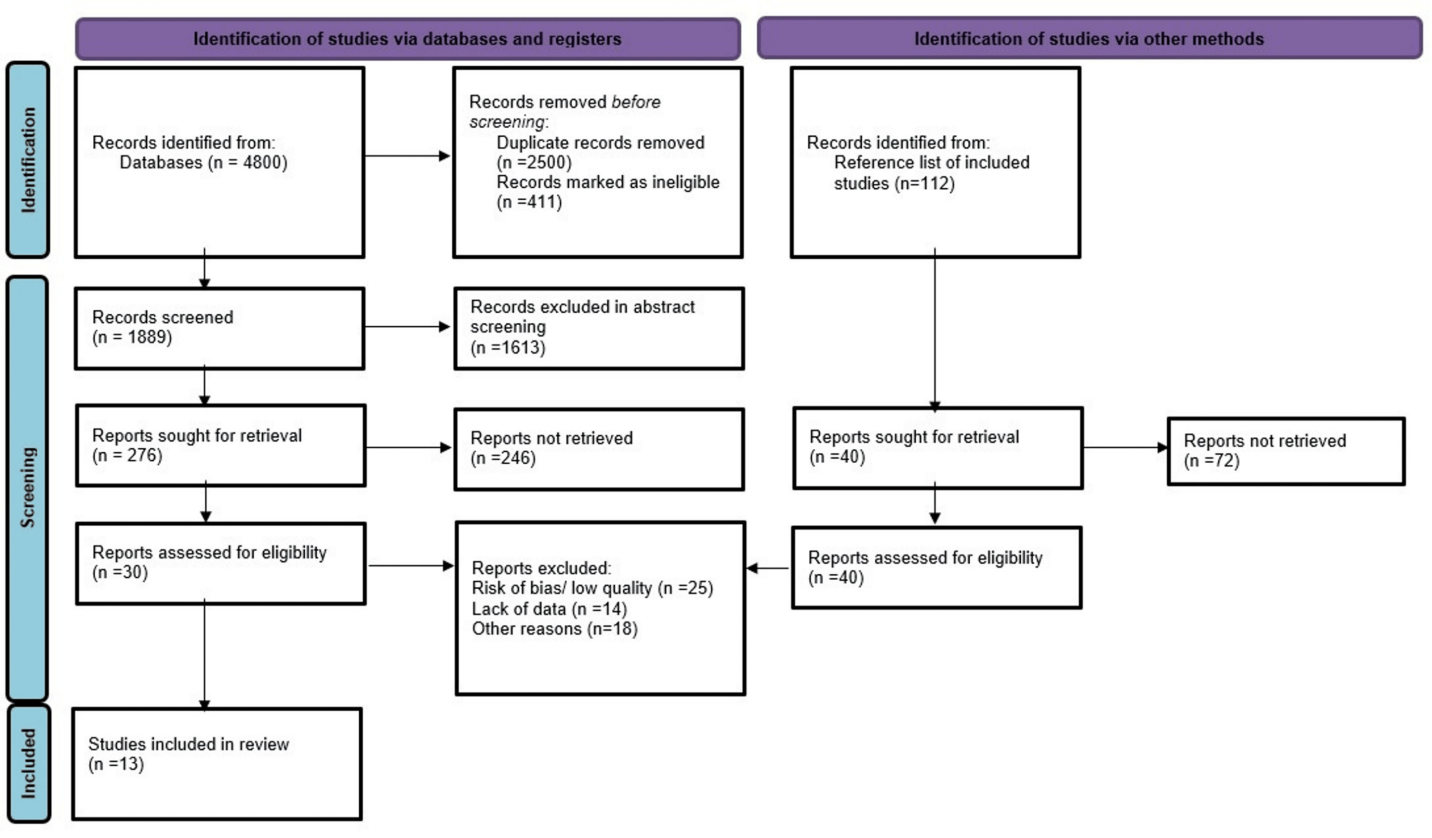
PRISMA flow diagram describing inclusion and exclusion criteria PRISMA: Preferred Reporting Items for Systematic Reviews and Meta-Analyses

All identified articles were screened independently by two reviewers based on titles and abstracts, followed by full-text review for eligibility. Disagreements were resolved by consensus or arbitration with a third reviewer. Reasons for study exclusion were documented systematically at the full-text review stage. The selection process was summarized in the PRISMA flow diagram (see Figure 1).

Data Extraction

Data were extracted independently by three reviewers using a standardized extraction form. Extracted variables included first author, year of publication, country, study design, sample size, mean age of participants, diagnostic criteria for PCOS, assay methods for TNF-α measurement, and reported outcomes (mean, SD, or median with IQRs). Where required, corresponding authors were contacted for missing data (see Table 1).

**Table 1:** Characteristics of all the studies included in the meta-analysis PCOS: polycystic ovary syndrome; SD: standard deviation; BMI: body mass index; TNF-α: tumor necrosis factor-alpha; ELISA: enzyme-linked immunosorbent assay; AES: Androgen Excess Society; HOMA: homeostatic model assessment; IL-6: interkeukin-6; WHR: waist-to-hip ratio Source: [8-20]

Study	Country	Design	N_PCOS	N_Controls	Age_PCOS (mean ± SD)	Age_Control (mean ± SD)	BMI_PCOS (mean ± SD)	BMI_Control (mean ± SD)	Diagnostic criteria	TNF_PCOS in pg/mL, mean ± SD	TNF_Control in pg/mL, mean ± SD	Method	Main finding reported
Goswami et al. [8]	India	Case-control	30	30	22.20 ± 8.32	27.60 ± 4.51	27.48 ± 4.41	24.18 ± 2.08	Rotterdam 2003	21.56 ± 11.15	16.73 ± 6.50	ELISA	TNF-α is significantly higher in PCOS and did not correlate with BMI, WHR, insulin resistance, or testosterone
Vasyukova et al. [9]	Russia	Case-control	98	61	26.1 ± 5.4	27.0 ± 5.1	28.4 ± 6.1	25.6 ± 5.3	Rotterdam 2003	7.24 ± 3.11	6.85 ± 2.96	ELISA	The levels of TNF-α increased in women with PCOS due to the difference in the lean subgroup
Thathapudi et al. [10]	India	Case-control	204	204	28.0 ± 3.6	28.0 ± 5.1	27.12 ± 4.93	23.40 ± 3.20	AES 2006	13.24 ± 10.0	5.50 ± 3.8	ELISA	All the PCOS had elevated body mass index, waist circumference, waist-to-hip ratio, fasting insulin, HOMA score, and serum TNF-α when compared with controls (p < 0.05)
Jabarpour et al. [11]	Iran	Case-control	26	27	30.42 ± 4.69	31.19 ± 4.57	26.08 ± 1.89	26.55 ± 1.89	Rotterdam 2003	19.25 ± 2.57	18.81 ± 1.78	ELISA	The TNF-α is high in PCOS subjects
Ha et al. [12]	China	Case-control	80	82	26.85 ± 3.82	27.63 ± 3.05	23.48 ± 2.87	21.71 ± 2.65	Rotterdam 2003	34.59 ± 12.21	19.61 ± 5.96	ELISA	The TNF-α levels in the patients with PCOS were higher than those in the control group (p < 0.01)
Al-Musawy et al. [13]	Iraq	Case-control	73	73	27.7 ± 5.8	29.4 ± 5.7	27.0 ± 3.4	26.0 ± 3.3	AES 2006	31.9 ± 67.1	10.7 ± 23.8	ELISA	TNF-alpha were elevated in PCOS women, and conclude that serum levels TNF-alpha are highly statistically significance in PCOS women than in healthy control group
Oróstica et al. [14]	Chile	Case-control	14	14	25.8 ± 2.7	26.6 ± 5.6	25.6 ± 0.2	22.4 ± 2.1	AES 2006	4.52 ± 0.48	4.73 ± 0.25	ELISA	TNF-α levels were similar between groups
Rahimi et al. [15]	Iran	Case-control	110	115	28.4 ± 5.7	29.1 ± 6.1	26.7 ± 4.9	25.9 ± 4.7	Rotterdam 2003	44.3 ± 17.2	37.9 ± 15.6	ELISA	TNF-α levels were high in PCOS groups
Barcellos et al. [16]	Brazil	Case-control	25	23	25.3 ± 1.1	26.8 ± 1.3	24.5 ± 3.9	23.8 ± 3.7	Rotterdam 2003	3.4 ± 4.8	2.1 ± 2.4	Multiplex-based immunoassay	Levels of TNF-Į were similar between the obese group and the lean group (2.1 vs. 1.9 pg/mL, p = 0.444)
Souza Dos Santos et al. [17]	Brazil	Case-control	20	20	28.5 ± 5.8	28.5 ± 5.8	25.0 ± 3.6	25.0 ± 3.6	Rotterdam 2003	9.06 ± 5.99	7.24 ± 2.44	Chemiluminescence	There were no differences in TNF-α and IL-6 levels between groups
Albayati and Abdulhameed [18]	Iraq	Case-control	65	60	30.17 ± 5.17	29.93 ± 5.53	29.22 ± 5.11	28.66 ± 5.90	Rotterdam 2003	75.99 ± 15.99	57.46 ± 12.00	ELISA	Significant differences were found in the serum levels of TNF-α between the two groups
Zafari Zangeneh et al. [19]	Iran	Case-control	85	86	27.1 ± 4.4	29.5 ± 5.1	27.39 ± 3.94	25.41 ± 3.76	Rotterdam 2003	10.91 ± 26.42	45.60 ± 172.48	ELISA	TNF-α plasma levels were high in PCOS groups
Cardoso et al. [20]	Brazil	Case-control	60	60	35 ± 7	33 ± 7	25.5 ± 3.0	26.3 ± 3.0	Rotterdam 2003	5.36 ± 1.95	4.41 ± 2.40	Chemiluminescence	TNF-α is higher in PCOS women and showed a direct relationship with increased body fat percentage

Quality Assessment

Risk of bias for included studies was assessed using the Newcastle-Ottawa Scale (NOS) for observational studies, with scoring across three domains: selection, comparability, and outcome assessment. All studies included in the final synthesis were of moderate to high quality, with low risk of bias.

Statistical Analysis

All statistical analyses were performed in R (version 4.2.2; R Foundation for Statistical Computing, Vienna, Austria). The primary outcome was the standardized mean difference (SMD) in circulating TNF-α levels between PCOS and control groups. When studies reported TNF-α as medians and IQRs, means and SDs were estimated using the Meta-Analysis Accelerator tool.

A random-effects model was prespecified to account for expected clinical and methodological heterogeneity. We included all eligible studies to obtain an overall average effect, consistent with methodological standards for observational meta-analyses. Missing SDs were estimated from reported standard errors (SEs) or 95% confidence intervals (CIs) when available; no data imputation was performed for TNF-α values. Meta-regression was conducted with caution due to the limited number of studies, acknowledging that statistical power to detect moderator effects is restricted when fewer than 10-15 studies are available. Statistical heterogeneity was quantified using Cochran’s Q test and the I² statistic, with I² values <25% considered low, 25%-50% moderate, and >75% high heterogeneity.

Assessment of Publication Bias

Potential publication bias was evaluated using both graphical and statistical approaches. Funnel plots were visually inspected for asymmetry, and Egger’s linear regression test was applied, with a p value of <0.05 indicating possible small-study effects. In addition, the trim-and-fill method (L-estimator) was used to estimate the number of missing studies and provide adjusted pooled estimates. Analyses were conducted using the meta and metafor packages in R.

Subgroup Analysis

Subgroup analyses were also performed according to 1) BMI, 2) diagnostic definitions applied, 3) assay method, and 4) country. To assess whether obesity status influenced TNF-α levels in PCOS, studies were stratified according to the mean BMI of PCOS cohorts: lean (mean BMI < 25 kg/m²) and obese (mean BMI ≥ 25 kg/m²). Subgroup-specific pooled SMDs with 95% CIs were calculated using random-effects models. Between-subgroup differences were evaluated with the χ²-based Q statistic.

For subgroup analyses according to the applied diagnostic definitions, studies were classified as using either the Rotterdam 2003 criteria or the AES 2006 criteria. Random-effects models were applied within each subgroup, and χ² tests were used to assess subgroup differences.

To examine whether laboratory techniques influenced pooled results, studies were stratified according to TNF-α measurement method: enzyme-linked immunosorbent assay (ELISA), chemiluminescence-based immunoassays, or multiplex-based immunoassays. Random-effects models were applied separately within each subgroup, and χ² tests were used to evaluate subgroup differences.

To explore geographic variability, studies were grouped by country of origin (India, Russia, Iran, China, Iraq, Chile, and Brazil). Random-effects models were fitted for each subgroup, and between-country differences were tested using the χ²-based Q statistic. Heterogeneity within subgroups was quantified using τ², τ, and I² statistics.

Sensitivity Analysis

The robustness of pooled estimates was examined using a leave-one-out sensitivity analysis, in which each study was sequentially excluded and the meta-analysis recalculated. This procedure assessed the influence of individual studies on overall results. Analyses were performed using the metainf() function in the meta package, and forest plots were generated to visualize effect stability.

Meta-Regression Analyses

To explore potential sources of heterogeneity, mixed-effects meta-regression analyses were performed using the metareg() function in the meta package in R. Continuous moderators examined in these models included the mean BMI of PCOS participants, the mean age of PCOS participants, and the total sample size across PCOS and control groups. For each moderator, regression coefficients, SEs, z-values, p values, and 95% CIs were calculated. The explanatory power of each moderator was quantified using the proportion of between-study heterogeneity accounted for (R²) [4,7].

## Review

Results

Study Characteristics

A total of 13 studies, encompassing 1,745 participants (890 women with PCOS and 855 controls), were included in the quantitative synthesis. Sample sizes per arm ranged from 14 to 204 participants. Regarding diagnostic criteria, 10 studies applied the Rotterdam 2003 criteria, while three studies used the Androgen Excess and PCOS Society (AES 2006) criteria. None of the included studies used the NIH 1990 criteria (see Table 1).

With respect to laboratory methods, 11 studies measured serum or uterine fluid TNF-α concentrations using ELISA, while two studies employed alternative methods, namely chemiluminescence immunoassay or multiplex-based immunoassay. The included studies were conducted across eight countries: India, Iran, Iraq, Russia, China, Brazil, Chile, and Turkey (see Table 1).

Pooled Effect Estimates

**Figure 2:**
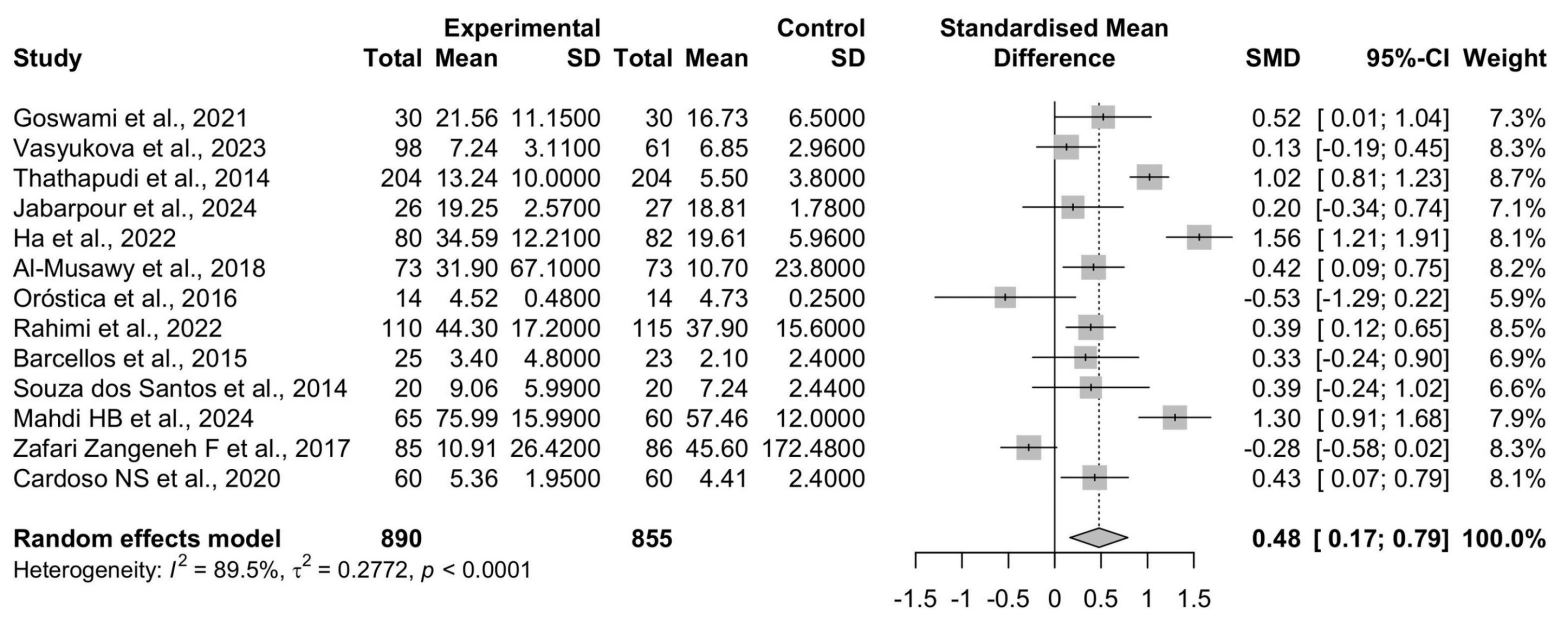
Forest plot of the random-effects meta-analysis comparing circulating TNF-α levels between women with PCOS and healthy controls. Each horizontal line represents the 95% CI for the SMD of an individual study, with the square size proportional to study weight. The diamond represents the pooled estimate. Overall, TNF-α levels were significantly higher in women with PCOS compared to controls (SMD = 0.48, 95% CI: 0.17-0.79, p = 0.0026). Substantial heterogeneity was observed across studies (I² = 89.5%, τ² = 0.2772, p < 0.0001) TNF-α: tumor necrosis factor-alpha; PCOS: polycystic ovary syndrome; CI: confidence interval; SMD: standardized mean difference Source: [8-20]

The random-effects meta-analysis demonstrated that women with PCOS had higher circulating TNF-α levels compared with healthy controls (SMD = 0.48, 95% CI = 0.17-0.79; z = 3.01; p = 0.0026). Effect sizes ranged from a negative association (SMD = -0.53, 95% CI = -1.29 to 0.22) to a strong positive association (SMD = 1.56, 95% CI = 1.21-1.91). Most studies reported a positive direction of association (see Figure 2).

Funnel Plot

Between-study heterogeneity was substantial (τ² = 0.2772, I² = 89.5%, p < 0.0001), reflecting considerable variability in effect size estimates across the included studies. To evaluate potential publication bias, a funnel plot was generated (Figure 2). Each circle represents an individual study plotted according to its SMD (X-axis) and standard error (Y-axis). The vertical line corresponds to the pooled effect estimate, while the diagonal lines denote pseudo 95% confidence limits.

**Figure 3:**
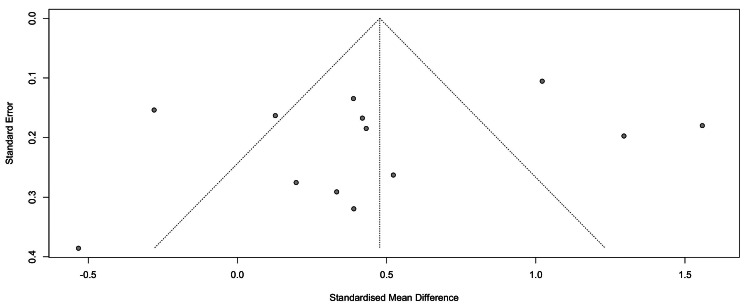
Funnel plot assessing potential publication bias among the included studies evaluating TNF-α levels in women with PCOS compared with healthy controls. Each circle represents an individual study, plotted by SMD on the X-axis and standard error on the Y-axis. The vertical line indicates the pooled effect size, and the diagonal lines represent pseudo 95% confidence limits. Visual inspection revealed no substantial asymmetry, suggesting a low likelihood of publication bias TNF-α: tumor necrosis factor-alpha; PCOS: polycystic ovary syndrome; SMD: standardized mean difference

Visual inspection revealed a relatively symmetrical distribution of studies around the pooled effect size. Although some scatter was evident, particularly among smaller studies with higher standard errors, there was no pronounced asymmetry. This pattern suggests that the overall synthesis is unlikely to be strongly influenced by the selective publication of positive findings (see Figure 3).

Moreover, Egger’s regression test did not reveal significant evidence of publication bias (t = -0.96, df = 11, p = 0.36). However, the presence of high heterogeneity indicates that other factors, such as differences in diagnostic criteria (Rotterdam vs. AES), population characteristics, or assay methodology, likely contributed to variability across studies.

Funnel Plot: Trim-and-Fill Analysis

**Figure 4:**
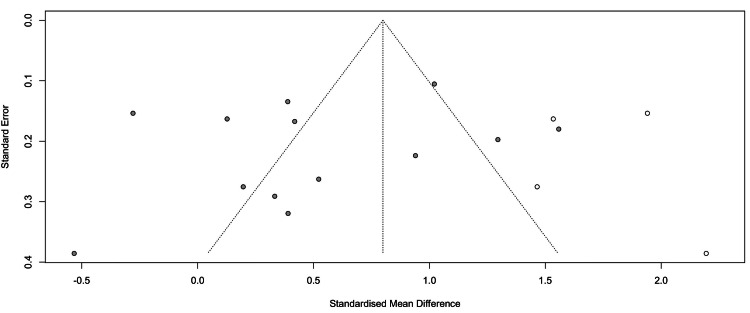
Funnel plot of studies evaluating TNF-α levels in women with PCOS compared with controls. Circles represent individual study effect sizes (SMDs) plotted against their standard errors. The distribution appears relatively symmetrical around the pooled effect estimate, and Egger’s test did not indicate significant publication bias. Circles in white indicate the imputed studies TNF-α: tumor necrosis factor-alpha; PCOS: polycystic ovary syndrome; SMD: standardized mean difference

Application of the trim-and-fill method imputed four potentially missing studies, which adjusted the pooled SMD upward from 0.48 (95% CI = 0.17-0.79) to 0.80 (95% CI = 0.44-1.16, p < 0.0001) (Figure 4). This suggests that small-study effects may have influenced the observed data. Nevertheless, since heterogeneity remained high (I² = 92.9%) and Egger’s test was nonsignificant, the original pooled estimate was deemed the more conservative and reliable summary of TNF-α differences between PCOS cases and controls (see Figure 4).

Subgroup Analysis by BMI

**Figure 5:**
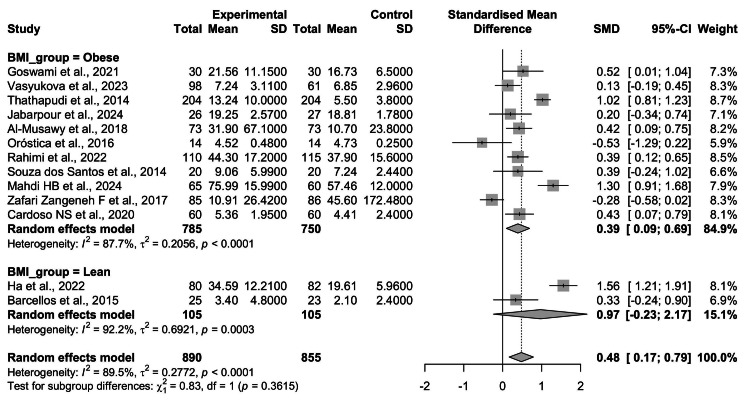
Subgroup analysis of circulating TNF-α levels in women with PCOS vs. healthy controls, stratified by BMI. The obese subgroup (11 studies) demonstrated a significant elevation in TNF-α among PCOS patients compared with obese controls (SMD = 0.39, 95% CI: 0.09-0.69, p < 0.01), whereas the lean subgroup (two studies) showed a larger but nonsignificant effect (SMD = 0.97, 95% CI: -0.23 to 2.17, p = 0.11). Between-subgroup differences were not statistically significant (p = 0.36) TNF-α: tumor necrosis factor-alpha; PCOS: polycystic ovary syndrome; BMI: body mass index; CI: confidence interval; SMD: standardized mean difference Source: [8-20]

To explore the influence of obesity on inflammatory status, studies were stratified into obese (k = 11) and lean (k = 2) cohorts (Figure 5). In the obese subgroup, women with PCOS exhibited significantly higher circulating TNF-α levels compared with obese controls (SMD = 0.39, 95% CI = 0.09-0.69; p < 0.01). However, heterogeneity remained substantial (I² = 87.7%), indicating variability across studies despite a consistent direction of effect.

In the lean subgroup, the pooled effect was larger (SMD = 0.97, 95% CI = -0.23 to 2.17; p = 0.11), but the association did not reach statistical significance, likely due to the small number of studies (n = 2) and very high heterogeneity (I² = 92.2%). A test for between-group differences revealed no significant moderation by BMI (Q = 0.83, df = 1, p = 0.36) (see Figure 5).

Taken together, these findings suggest that TNF-α elevation is more consistently observed in obese women with PCOS, reinforcing the role of adiposity in amplifying inflammatory responses.

Subgroup Analysis by Diagnostic Criteria

**Figure 6:**
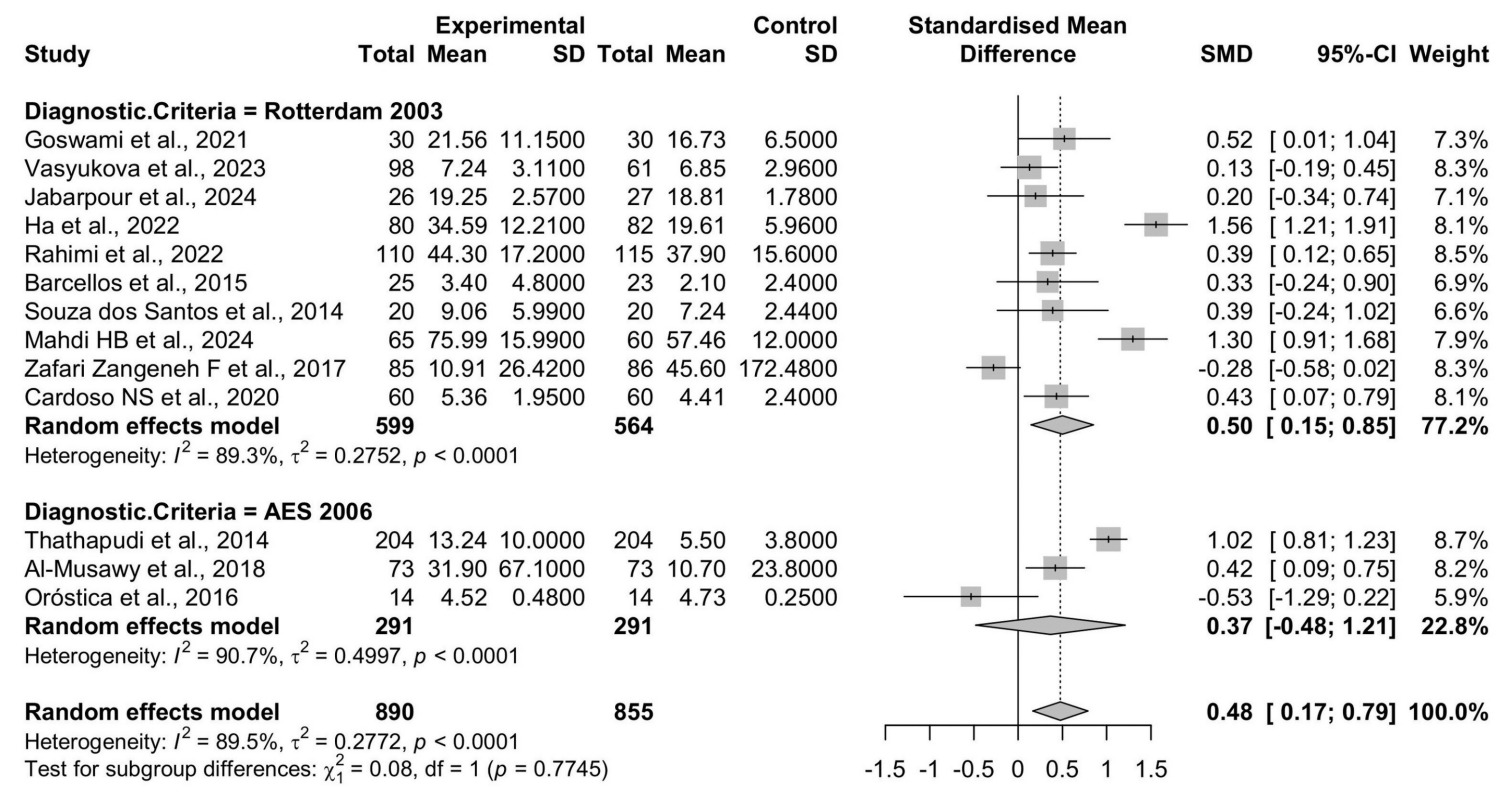
Subgroup analysis of circulating TNF-α levels in women with PCOS versus healthy controls, stratified by diagnostic criteria. Studies employing the Rotterdam 2003 definition (k = 10) demonstrated a significant elevation in TNF-α levels among PCOS patients (SMD = 0.50, 95% CI: 0.15-0.85, p < 0.01), with substantial heterogeneity (I² = 89.3%). In contrast, studies using the AES 2006 criteria (k = 3) showed a nonsignificant pooled effect (SMD = 0.37, 95% CI: -0.48 to 1.21, p = 0.39), also with high heterogeneity (I² = 90.7%). The test for subgroup differences indicated no statistically significant variation between diagnostic definitions (p = 0.77) TNF-α: tumor necrosis factor-alpha; PCOS: polycystic ovary syndrome; CI: confidence interval; SMD: standardized mean difference; AES: Androgen Excess Society Source: [8-20]

A subgroup analysis was performed to investigate whether the choice of diagnostic criteria for PCOS influenced the observed association with TNF-α levels (Figure 6). In studies applying the Rotterdam 2003 criteria (k = 10), pooled results demonstrated a significant elevation of TNF-α levels in women with PCOS compared with controls (SMD = 0.50, 95% CI = 0.15-0.85; p < 0.01). However, heterogeneity was considerable (I² = 89.3%), reflecting methodological and population variability across studies.

In contrast, studies using the AES 2006 criteria (k = 3) yielded a nonsignificant pooled effect (SMD = 0.37, 95% CI = -0.48 to 1.21; p = 0.39), with similarly high heterogeneity (I² = 90.7%) (see Figure 6).

The test for subgroup differences showed no statistically significant variation between diagnostic definitions (Q = 0.08, df = 1, p = 0.77). This indicates that while elevated TNF-α levels are more consistently reported in studies adopting the broader Rotterdam criteria, diagnostic classification alone does not account for the between-study heterogeneity.

Subgroup Analysis by Assay Method

**Figure 7:**
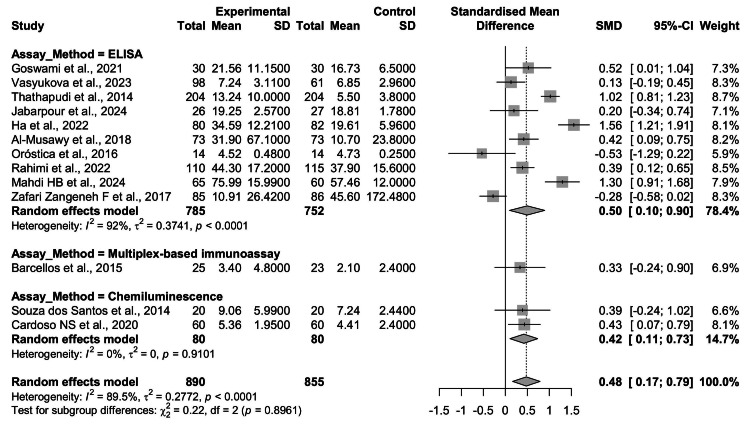
Subgroup analysis of circulating TNF-α levels in women with PCOS vs. controls, stratified by assay method. Studies using ELISA (k = 10) showed a significant elevation in TNF-α among PCOS patients (SMD = 0.50, 95% CI: 0.10-0.90, p < 0.05), although heterogeneity was substantial (I² = 92%). Chemiluminescence-based assays (k = 2) also demonstrated a significant pooled effect (SMD = 0.42, 95% CI: 0.11-0.73, p < 0.01) with no heterogeneity (I² = 0%). A single study using multiplex-based immunoassay reported a nonsignificant effect (SMD = 0.33, 95% CI: -0.24 to 0.90). The test for subgroup differences was not significant (p = 0.90), suggesting that assay methodology did not account for the observed heterogeneity TNF-α: tumor necrosis factor-alpha; PCOS: polycystic ovary syndrome; ELISA: enzyme-linked immunosorbent assay; CI: confidence interval; SMD: standardized mean difference Source: [8-20]

Given the variation in laboratory techniques used to quantify TNF-α, subgroup analysis was conducted based on assay methodology (Figure 7). Studies employing ELISA (k = 10) reported a significant pooled effect, with higher TNF-α levels in PCOS compared with controls (SMD = 0.50, 95% CI = 0.10-0.90; p < 0.05). However, heterogeneity was substantial (I² = 92%), suggesting methodological or population-level differences despite a consistent overall direction.

In contrast, chemiluminescence-based assays (k = 2) demonstrated a significant association with moderate effect size (SMD = 0.42, 95% CI = 0.11-0.73; p < 0.01), and importantly, showed no heterogeneity (I² = 0%), indicating high consistency between the two studies. The single study utilizing a multiplex immunoassay (k = 1) yielded a nonsignificant result (SMD = 0.33, 95% CI = -0.24 to 0.90).

The test for subgroup differences was not statistically significant (Q = 0.22, df = 2, p = 0.90), indicating that assay methodology did not explain between-study heterogeneity. Nevertheless, the stability of chemiluminescence-based findings, in contrast with the variability among ELISA studies, highlights potential differences in assay reproducibility and standardization (see Figure 7).

Country-Based Subgroup Analysis

**Figure 8:**
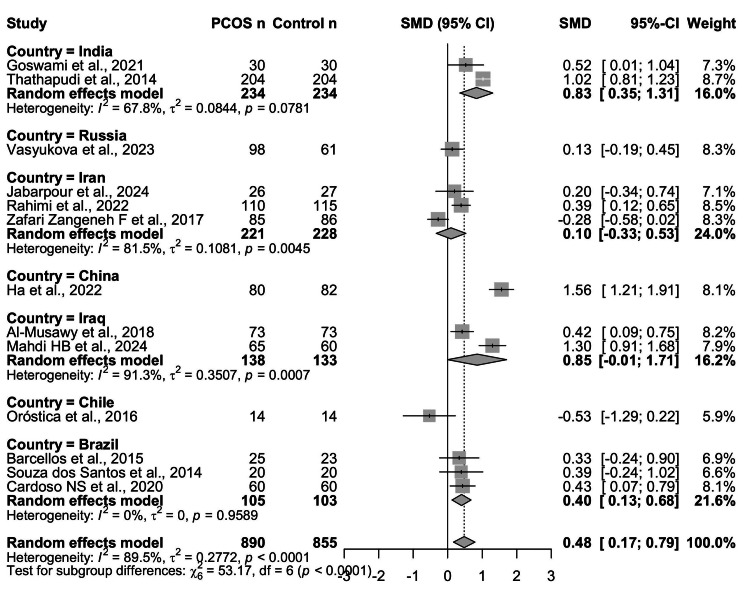
Subgroup analysis of circulating TNF-α levels in women with PCOS vs. controls, stratified by country of origin. Significant elevations in TNF-α were observed in studies from India (SMD = 0.83, 95% CI: 0.35-1.31) and China (SMD = 1.56, 95% CI: 1.21-1.91), with moderate heterogeneity in the Indian subgroup (I² = 67.8%). Studies from Iraq also showed a borderline significant pooled effect (SMD = 0.85, 95% CI: -0.01 to 1.71) with very high heterogeneity (I² = 91.3%). In contrast, studies from Iran (SMD = 0.10, 95% CI: -0.33 to 0.53) and Chile (SMD = -0.53, 95% CI: -1.29 to 0.22) reported nonsignificant associations. Brazilian studies demonstrated a moderate, consistent effect (SMD = 0.40, 95% CI: 0.13-0.68) with no heterogeneity (I² = 0%). A single Russian study showed a small, nonsignificant effect (SMD = 0.13, 95% CI: -0.19 to 0.45). The test for subgroup differences was statistically significant (Q = 53.17, df = 6, p < 0.0001), indicating that country of origin moderated the observed association between PCOS and TNF-α levels TNF-α: tumor necrosis factor-alpha; PCOS: polycystic ovary syndrome; CI: confidence interval; SMD: standardized mean difference Source: [8-20]

To investigate whether geographic region influenced the association between PCOS and TNF-α, a country-based subgroup analysis was performed (Figure 8). Studies conducted in India (k = 2) reported a significant pooled effect, with elevated TNF-α levels among women with PCOS (SMD = 0.83, 95% CI = 0.35-1.31), though heterogeneity was moderate (I² = 67.8%). A single study from China (k = 1) showed a large and highly significant effect (SMD = 1.56, 95% CI = 1.21-1.91) [12].

In Iraq (k = 2), the pooled analysis indicated a borderline significant increase in TNF-α (SMD = 0.85, 95% CI = -0.01 to 1.71), but heterogeneity was very high (I² = 91.3%), limiting interpretability. Conversely, studies from Iran (k = 3) demonstrated no significant association (SMD = 0.10, 95% CI = -0.33 to 0.53; I² = 81.5%), and the single study from Chile (k = 1) suggested a negative but nonsignificant effect (SMD = -0.53, 95% CI: -1.29 to 0.22). Findings from Brazil (k = 3) revealed a moderate, consistent association (SMD = 0.40, 95% CI = 0.13-0.68) with no heterogeneity (I² = 0%), while the lone study from Russia (k = 1) reported a small, nonsignificant effect (SMD = 0.13, 95% CI = -0.19 to 0.45).

The test for subgroup differences was statistically significant (Q = 53.17, df = 6, p < 0.0001), indicating that country of origin contributed meaningfully to between-study heterogeneity. These findings suggest that both population-specific factors (e.g., ethnicity, lifestyle, and comorbidities) and methodological differences may underlie the geographic variability in reported TNF-α levels in PCOS (see Figure 8).

Sensitivity Analysis

**Figure 9:**
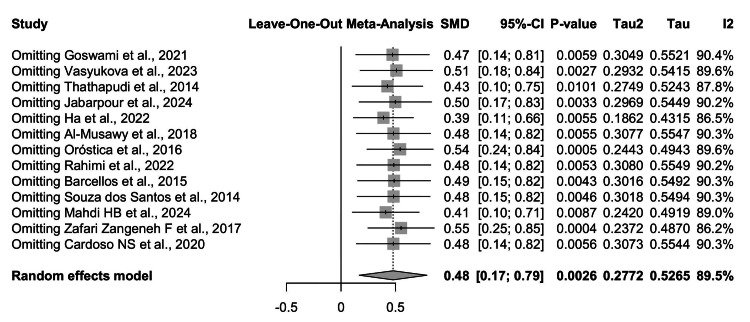
Leave-one-out sensitivity analysis of circulating TNF-α levels in women with PCOS compared to controls. Sequential omission of each study did not materially alter the pooled effect size, with SMDs consistently ranging from 0.39 to 0.55 and all 95% CIs excluding the null. Heterogeneity (I² = 86%-90%) remained substantial across iterations, indicating that no single study disproportionately influenced the overall findings. These results confirm the robustness and stability of the observed association between PCOS and elevated TNF-α levels TNF-α: tumor necrosis factor-alpha; PCOS: polycystic ovary syndrome; CI: confidence interval; SMD: standardized mean difference Source: [8-20]

To evaluate the robustness of the findings, a leave-one-out sensitivity analysis was performed, sequentially omitting each individual study from the pooled model (Figure 9).

Across all iterations, the pooled SMD remained statistically significant, ranging between 0.39 and 0.55, with 95% CIs consistently excluding the null. The exclusion of Ha et al. [12] and Albayati and Abdulhameed [18] slightly attenuated the overall effect size, whereas the omission of Oróstica et al. [14] and Zafari Zangeneh et al. [19] increased the pooled estimate (see Figure 9).

Despite these minor variations, the direction and magnitude of the association remained stable, underscoring the consistency of the observed effect. Importantly, heterogeneity persisted at high levels (I² = 86%-90%) across all iterations, suggesting that variability is not attributable to any single study but rather reflects broader differences in study populations, methodologies, or diagnostic approaches. These results provide strong evidence that the association between PCOS and elevated TNF-α levels is robust and not driven by outlier studies.

Meta-Regression by BMI

**Figure 10:**
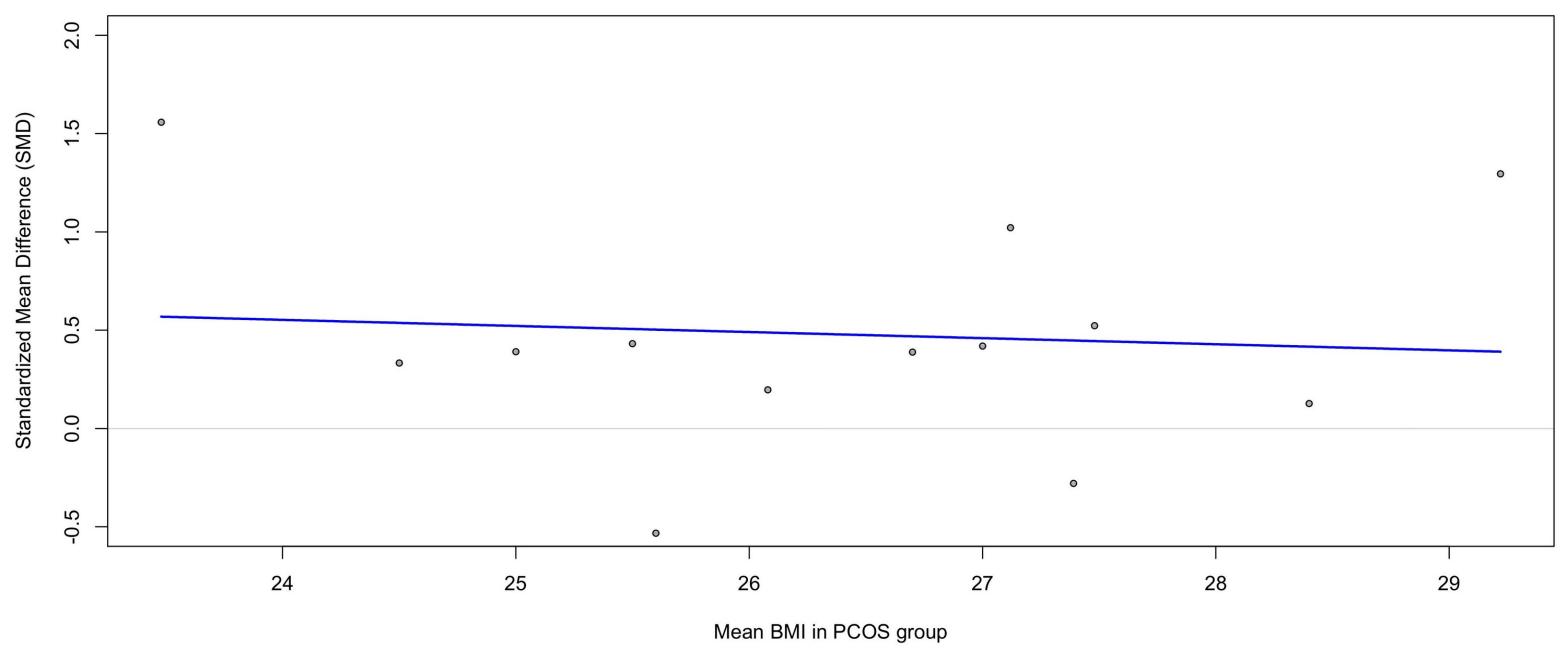
Meta-regression analysis examining the association between the mean BMI of PCOS participants and SMDs in circulating TNF-α levels. The regression slope was negative but not statistically significant (β = -0.031, p = 0.77), indicating that mean BMI did not explain the heterogeneity of effect sizes across studies. The bubble plot shows study-level effect sizes with a regression line (blue) fitted under a mixed-effects model TNF-α: tumor necrosis factor-alpha; PCOS: polycystic ovary syndrome; SMD: standardized mean difference; BMI: body mass index Source: [8-20]

To further evaluate the role of adiposity in moderating the association between PCOS and TNF-α, a meta-regression was conducted using the mean BMI of PCOS participants as a covariate (Figure 10). The regression slope was negative but not statistically significant (β = -0.031, SE = 0.107, p = 0.77; 95% CI = -0.24 to 0.18). This indicates that higher BMI did not systematically predict larger effect sizes across studies. The model explained no between-study variance (R² = 0%), and residual heterogeneity remained high (τ² = 0.30, I² ≈ 90%) (see Figure 10).

These findings suggest that while obesity contributes to elevated TNF-α levels at the clinical level, as seen in subgroup analyses, the variation in mean BMI across studies does not explain the heterogeneity observed in this meta-analysis. Other factors, such as diagnostic definitions, assay variability, and regional differences, are likely to underlie the observed inconsistencies.

Meta-Regression by Age

**Figure 11:**
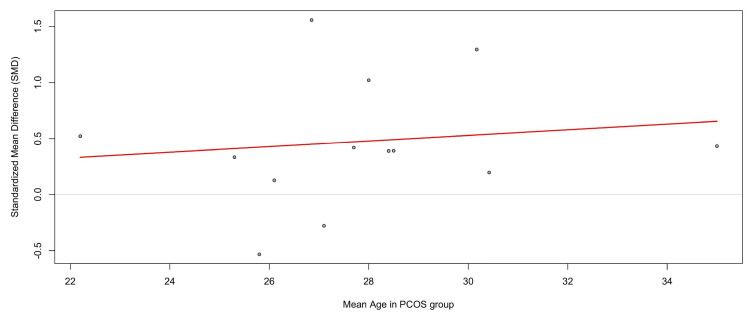
Meta-regression of SMDs in circulating TNF-α levels between PCOS patients and controls, with the mean age of PCOS cohorts as a moderator. The regression line (red) indicates a nonsignificant positive trend (β = 0.025, p = 0.65). The wide scatter of effect sizes across the 22-35 years age range, combined with high residual heterogeneity (I² ≈ 90%), suggests that age does not account for between-study variability TNF-α: tumor necrosis factor-alpha; PCOS: polycystic ovary syndrome; CI: confidence interval; SMD: standardized mean difference Source: [8-20]

To assess whether participant age influenced study outcomes, a meta-regression was conducted with the mean age of PCOS cohorts as the moderator (Figure 11). The analysis showed that mean age did not significantly moderate effect sizes (β = 0.025, 95% CI = -0.085 to 0.136; p = 0.65). The model explained none of the between-study variance (R² = 0%), and residual heterogeneity remained high (I² ≈ 90%) (see Figure 11).

Although the regression line indicated a slight positive slope, the wide scatter of effect sizes across the 22-35 year age range indicates that age was not a meaningful contributor to variability. These results suggest that differences in TNF-α levels between PCOS and controls are not dependent on the mean age of study participants.

Meta-Regression by Sample Size

**Figure 12:**
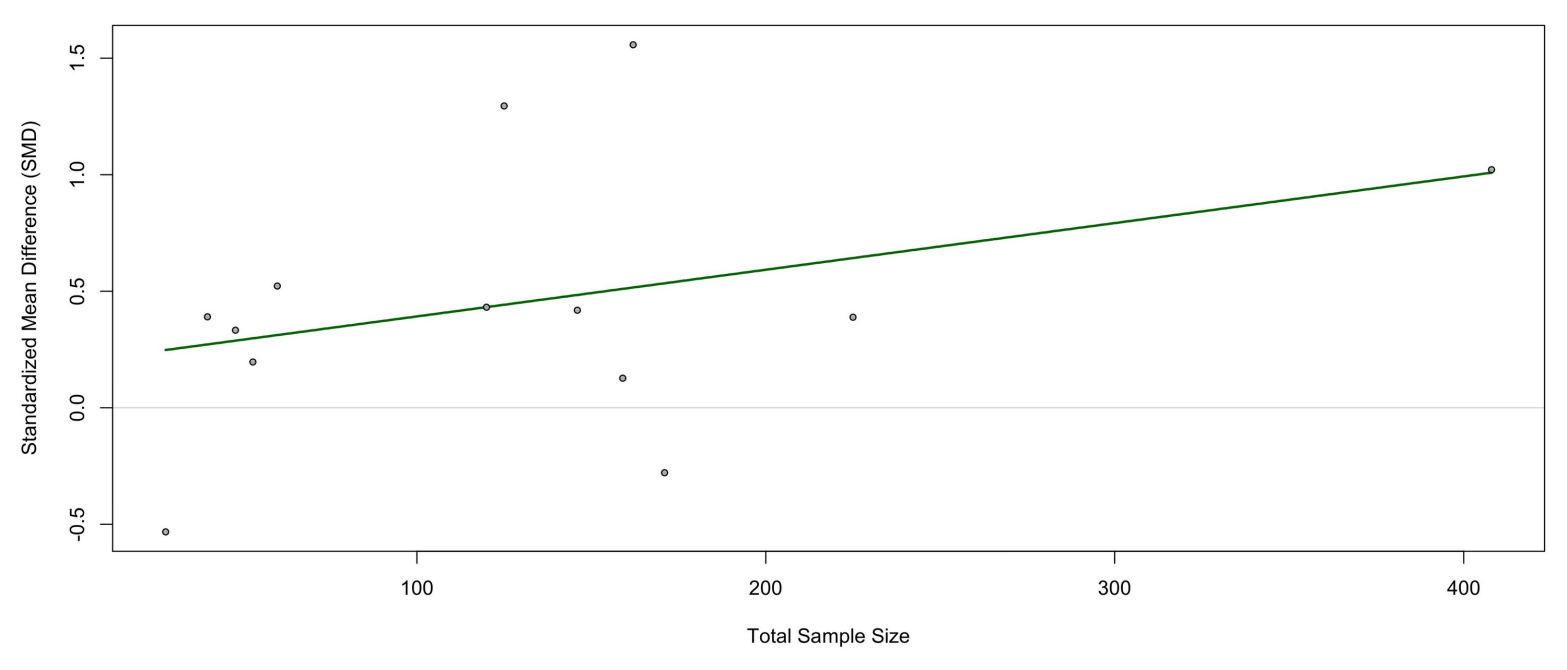
Meta-regression of SMDs in circulating TNF-α levels between PCOS patients and controls, with total study sample size as a moderator. The regression line (green) indicates a nonsignificant positive association (β = 0.0020, p = 0.20). Larger studies tended to report slightly higher effect sizes, but this relationship was not statistically significant. Residual heterogeneity remained high (I² = 87.5%), suggesting that sample size alone did not explain between-study variability TNF-α: tumor necrosis factor-alpha; PCOS: polycystic ovary syndrome; CI: confidence interval; SMD: standardized mean difference Source: [8-20]

To explore whether study precision influenced the magnitude of observed effects, a meta-regression was conducted with total sample size as the moderator (Figure 12). The analysis revealed a nonsignificant positive association between sample size and effect size (β = 0.0020, 95% CI = -0.0010 to 0.0050; p = 0.20). Although larger studies tended to report slightly higher SMDs, the trend did not achieve statistical significance.

The model explained only a small proportion of between-study heterogeneity (R² = 4.36%), while residual heterogeneity remained high (I² = 87.5%). This indicates that sample size did not meaningfully account for the observed variability across studies. These results suggest that the association between PCOS and TNF-α is consistent across both smaller and larger studies, and differences in study size are unlikely to explain the heterogeneity detected in the meta-analysis (see Figure 12).

Certainty of Evidence (Grading of Recommendations Assessment, Development, and Evaluation)

The certainty of evidence synthesized was evaluated using the Grading of Recommendations Assessment, Development and Evaluation framework. Thirteen case-control studies (n = 1,745; 890 PCOS, 855 controls) were included (see Table 2).

**Table 2:** The certainty of the evidence regarding circulating TNF-α levels in women with PCOS assessed using the GRADE TNF-α: tumor necrosis factor-alpha; SMD: standardized mean difference; CI: confidence interval; PCOS: polycystic ovary syndrome; GRADE: Grading of Recommendations Assessment, Development and Evaluation

Outcome	Studies (n)	Participants	Design	Risk of bias	Inconsistency	Indirectness	Imprecision	Publication bias	Pooled effect (random-effects)	Certainty
Circulating TNF-α (pg/mL)	13	1,745	Case–control	Not serious	Serious	Not serious	Not serious	Not serious	SMD = 0.48 (95% CI 0.17–0.79)	⊕◯◯◯ Very low

The overall risk of bias was judged to be low, as the majority of studies were rated as high quality by both the NOS (7-9/9 points; see the Appendix) with clear case definitions, appropriate control selection, and validated assays (ELISA, chemiluminescence) applied uniformly across groups. No serious concerns about indirectness were identified, since all studies directly compared TNF-α concentrations between PCOS patients diagnosed using internationally recognized criteria (Rotterdam 2003 or AES 2006) and healthy controls. Imprecision was also not considered a serious concern, as the pooled SMD was consistently positive (SMD = 0.48, 95% CI = 0.17-0.79), and leave-one-out sensitivity analyses confirmed statistical robustness, with all iterations remaining significant. However, some concerns were noted for inconsistency, given the substantial heterogeneity observed, which persisted despite multiple subgroup and meta-regression analyses (BMI, age, and sample size). Publication bias could not be excluded, as funnel plot asymmetry was suggested, although Egger’s test did not indicate strong small-study effects. Taken together, the certainty of evidence for this outcome was graded as low, downgraded for inconsistency and possible publication bias. Despite this, the direction of the effect was stable, supporting the conclusion that women with PCOS exhibit elevated TNF-α levels relative to controls, reinforcing the role of chronic inflammation in PCOS pathophysiology.

Discussion

The present meta-analysis of 13 studies (n = 1,745) found a modest but statistically significant elevation of circulating TNF-α in women with PCOS compared with healthy controls (SMD = 0.48; 95% CI = 0.17-0.79; p = 0.0026). This association persisted across sensitivity checks but was accompanied by very high between-study heterogeneity (I² ≈ 89.5%). Subgroup results showed a consistent, smaller effect in obese cohorts (SMD = 0.39) and larger, imprecise effects in the sparse lean cohort data (SMD = 0.97, nonsignificant). Assay-specific analyses produced broadly concordant findings (ELISA SMD ≈ 0.50; chemiluminescence SMD ≈ 0.42), and meta-regressions for mean BMI, age, and sample size did not explain the heterogeneity.

These results fit into a developing, but not uniform, literature. Escobar-Morreale et al. [21], using a conservative, pre-2010 evidence base and stringent inclusion rules, reported no significant pooled TNF-α increase; they also flagged possible dissemination bias that could have underestimated true differences. Gao et al. [5] synthesized a larger and partly different set of studies and reported a larger pooled SMD (≈0.60) with similarly high heterogeneity; when Gao et al. removed several influential studies, heterogeneity dropped, and the effect size attenuated. Taken together, the three syntheses present a coherent picture: TNF-α is frequently elevated in PCOS, particularly in settings characterized by greater insulin resistance, androgen excess, or coexisting obesity, but the effect magnitude varies by study population, phenotype mix, and laboratory methodology [5-7].

Our analysis advances the field in three ways. First, by updating the evidence base and applying robust random-effects estimation and multiple sensitivity approaches, we provide a conservative, reproducible summary estimate while transparently quantifying uncertainty. Second, we systematically identify and quantify moderators, most notably geographic origin and BMI strata, that explain some variation and should be reported and considered in future work. Third, by documenting persistent heterogeneity, we highlight specific reporting and methodological gaps that currently limit interpretability [8-10].

There are certain limitations our current synthesis has that warrant emphasis. The evidence base is dominated by cross-sectional observational studies, precluding causal inference. Reporting on key covariates (PCOS phenotype, fasting status, cycle phase, medications) was often incomplete, restricting covariate adjustment and individual participant data-level analyses. Assay heterogeneity and variable preanalytic protocols likely contributed materially to between-study variance. Finally, subgroup and meta-regression analyses were constrained by the moderate number of studies (k = 13), limiting statistical power to detect moderators [11-15].

Future research should 1) adopt standardized assay platforms and report kit/manufacturer, limits of detection and preanalytic procedures; 2) preregister phenotype-stratified biomarker studies with careful control or matching for BMI and medication use; 3) collect and share harmonized individual participant data to permit adjustment for adiposity, age, cycle phase, and treatments; and 4) prioritize prospective and intervention designs that can test whether TNF-α changes track causal pathways in PCOS [16-20].

In conclusion, our study supports a modest elevation of circulating TNF-α in PCOS populations while emphasizing that the magnitude and consistency vary by context. By clarifying where heterogeneity arises and what data are missing, this meta-analysis can help design more rigorous, phenotype-aware biomarker studies and inform sample size and assay choices for future mechanistic and interventional research.

## Conclusions

This systematic review and meta-analysis provide robust evidence that circulating TNF-α levels are modestly but significantly elevated in women with PCOS compared with healthy controls. These findings suggest that TNF-α may serve as a potential inflammatory biomarker reflective of metabolic dysregulation in PCOS, offering mechanistic insight into its pathophysiology. Future longitudinal and interventional studies should clarify causality, standardize methodologies, and explore whether modulation of TNF-α levels could serve diagnostic or therapeutic purposes in the management of PCOS.

## Appendices

**Table 3:** Quality assessment sheet (Newcastle-Ottawa Scale) BMI: body mass index Source: [8-20]

Study	Case definition adequate (0/1)	Representativeness of cases (0/1)	Selection of controls (0/1)	Definition of controls (0/1)	BMI/key confounders controlled (0/1)	Additional confounders controlled (0/1)	Ascertainment of exposure (0/1)	Same method for cases and controls (0/1)	Nonresponse rate reported (0/1)	Total score (0-9)	Quality (good/fair/poor)
Goswami et al. [8]	1	1	1	1	1	1	1	1	0	8	Good
Vasyukova et al. [9]	1	1	1	1	1	1	1	1	1	9	Excellent
Thathapudi et al. [10]	1	1	1	1	1	1	1	1	0	8	Good
Jabarpour et al. [11]	1	1	1	1	1	0	1	1	0	7	Good
Ha et al. [12]	1	1	1	1	1	0	1	1	0	7	Good
Al-Musawy et al. [13]	1	1	1	1	1	1	1	1	0	8	Good
Oróstica et al. [14]	1	1	1	1	1	1	1	1	0	8	Good
Rahimi et al. [15]	1	1	1	1	1	1	1	1	0	8	Good
Barcellos et al. [16]	1	1	1	1	1	1	1	1	0	8	Good
Souza Dos Santos et al. [17]	1	1	1	1	1	1	1	1	0	8	Good
Albayati and Abdulhameed [18]	1	1	1	1	1	1	1	1	0	8	Good
Zafari Zangeneh et al. [19]	1	1	1	1	1	1	1	1	1	9	Excellent
Cardoso et al. [20]	1	1	1	1	1	1	1	1	1	9	Good

## References

[1] Liu J, Wu Q, Hao Y, Jiao M, Wang X, Jiang S, Han L (2021). Measuring the global disease burden of polycystic ovary syndrome in 194 countries: Global Burden of Disease Study 2017. Hum Reprod.

[2] Athira PR, Swathi D, Sukesh N, Pillai A, Delna NS (2024). The intersection of hepcidin and polycystic ovary syndrome: a review of current understanding. Asian J Med Health.

[3] Hussein RS, Dayel SB, Abahussein O (2024). Polycystic ovary syndrome and reproductive health: a comprehensive review. Clin Exp Obstet Gynecol.

[4] Bansal B, Thazhuthadath Kishore A, Kathiresan S (2025). A systematic review of inflammatory markers in polycystic ovary syndrome (PCOS) and meta-analysis of interleukin-6 (IL-6) in case-control studies. Cureus.

[5] Gao L, Gu Y, Yin X (2016). High serum tumor necrosis factor-alpha levels in women with polycystic ovary syndrome: a meta-analysis. PLoS One.

[6] Sushitha ES, Shetty PP, Kathiresan S (2025). The role of leptin in pathology of polycystic ovary syndrome (PCOS). An Overview of Disease and Health Research.

[7] (2025). Tumor necrosis factor-alpha (TNF-α) levels in women with PCOS: a systematic review and meta-analysis of observational studies. https://osf.io/vtjer/overview.

[8] Goswami S, Choudhuri S, Bhattacharya B (2021). Chronic inflammation in polycystic ovary syndrome: a case-control study using multiple markers. Int J Reprod Biomed.

[9] Vasyukova E, Zaikova E, Kalinina O (2023). Inflammatory and anti-inflammatory parameters in PCOS patients depending on body mass index: a case-control study. Biomedicines.

[10] Thathapudi S, Kodati V, Erukkambattu J, Katragadda A, Addepally U, Hasan Q (2014). Tumor necrosis factor-alpha and polycystic ovarian syndrome: a clinical, biochemical, and molecular genetic study. Genet Test Mol Biomarkers.

[11] Jabarpour M, Amidi F, Aleyasin A, Nashtaei MS, Marghmaleki MS (2024). Randomized clinical trial of astaxanthin supplement on serum inflammatory markers and ER stress-apoptosis gene expression in PBMCs of women with PCOS. J Cell Mol Med.

[12] Ha LX, Li WX, Du YD, Yuan YY, Qu XX (2022). Tumor necrosis factor alpha level in the uterine fluid of patients with polycystic ovary syndrome and its correlation with clinical parameters. J Inflamm Res.

[13] Al-Musawy SH, Al-Saimary IE, Flaifil MS (2018). Levels of cytokines profile in polycystic ovary syndrome. Med J Babylon.

[14] Oróstica L, Astorga I, Plaza-Parrochia F (2016). Proinflammatory environment and role of TNF-α in endometrial function of obese women having polycystic ovarian syndrome. Int J Obes (Lond).

[15] Rahimi G, Shams S, Aslani MR (2022). Effects of crocin supplementation on inflammatory markers, lipid profiles, insulin and cardioprotective indices in women with PCOS: a randomized, double-blind, placebo-controlled trial. Phytother Res.

[16] Barcellos CR, Rocha MP, Hayashida SA, Dantas WS, Dos Reis Vieira Yance V, Marcondes JA (2015). Obesity, but not polycystic ovary syndrome, affects circulating markers of low-grade inflammation in young women without major cardiovascular risk factors. Hormones (Athens).

[17] Souza Dos Santos AC, Soares NP, Costa EC, de Sá JC, Azevedo GD, Lemos TM (2015). The impact of body mass on inflammatory markers and insulin resistance in polycystic ovary syndrome. Gynecol Endocrinol.

[18] Albayati HBM, Abdulhameed WA (2024). TNF-alpha and IL-10 levels in Iraqi PCOS and non-PCOS patients undergoing ICSI: an immunological perspective. Al-rafidain J Med Sci.

[19] Zafari Zangeneh F, Naghizadeh MM, Masoumi M (2017). Polycystic ovary syndrome and circulating inflammatory markers. Int J Reprod Biomed.

[20] Cardoso NS, Ribeiro VB, Dutra SG, Ferriani RA, Gastaldi AC, Araújo JE, Souza HC (2020). Polycystic ovary syndrome associated with increased adiposity interferes with serum levels of TNF-alpha and IL-6 differently from leptin and adiponectin. Arch Endocrinol Metab.

[21] Escobar-Morreale HF, Luque-Ramírez M, González F (2011). Circulating inflammatory markers in polycystic ovary syndrome: a systematic review and metaanalysis. Fertil Steril.

